# Tyrosine-kinase inhibition results in EGFR clustering at focal adhesions and consequent exocytosis in uPAR down-regulated cells of Head and Neck cancers

**DOI:** 10.1186/1476-4598-7-47

**Published:** 2008-06-03

**Authors:** Samah Abu-Ali, Abbas Fotovati, Kanemitsu Shirasuna

**Affiliations:** 1 Department of Oral and Maxillofacial Surgery, Graduate school of Dental Science, Kyushu University, Higashi-Ku, Fukuoka, Japan; 2 Japan Society for Promotion of Science, Chiyoda-Ku, Tokyo, Japan; 3 Department of Molecular Surgery, The research center for innovative cancer therapy, Kurume University, Kurume, Japan

## Abstract

**Background:**

Antisense (AS) induced down-regulation of uPAR in ACCS adenoid-cyctic carcinoma cells decreased the cellular adhesion and invasion on various extracellular matrices. Additionally, ACCS-AS cells showed an increased EGFR expression and other behavioral similarities to NA-SCC, a typical highly proliferative but less invasive squamous cell carcinoma (SCC) cell line of the head and neck. ACCS, ACCS-AS and NA-SCC cells were used to elucidate the relationships between uPAR down-regulation and EGFR inhibition.

**Results:**

Tyrosine kinase inhibitor Gefitinib (IRESSA, ZD 1839) significantly reduced the chemotactic cell migration and adhesion. This was associated with reduced EGFR and ERK activation. In addition, anti-proliferative effect of gefitinib in uPAR down-regulated ACCS-AS was significantly higher than parental ACCS, to levels comparable to gefitinib-sensitive NA-SCC cells. This was evidenced by both reduced dosage and duration of treatment. Furthermore, time-lapse videography showed that treatment with gefitinib was also associated with cell rounding and loss of pseudopodia, mostly in ACCS-AS rather than parental ACCS cells. There were also evidences of formation and exocytosis of vacuole-like structures in ACCS-AS, as well as NA-SCC, but not in parental ACCS cells. Interestingly, immunocytochemistry showed that the exocytotic vacuoles actually contained de-activated EGFR.

**Conclusion:**

Our results suggested that down-regulation of uPAR affected the fate of EGFR in high EGFR expressing cells. Furthermore, combining the uPAR down-regulation with EGFR inhibition showed a synergistic anti-tumor effect and might provide an alternative method to increase anti-proliferative effect of tyrosine kinase inhibitors with lower doses and duration to reduce their side effects during cancer control.

## Background

Urokinase Plasminogen Activator Receptor (uPAR) is a three-domain glycoprotein linked to the cell membrane by a glycosylphosphatidylinositol. It facilitates cellular movement, providing a proper condition for tumor-cell invasion, chemotaxis, and cellular adhesion [1,2]. Down-regulation of uPAR by using antisense (AS) or gene-therapy approaches has increased survival in animal models of cancer [3,4]. The stable transfection of uPAR antisense to glioblastoma clones resulted in an inability of the cells to generate tumors when transplanted into nude mice [5] and reduced invasiveness *in-vitro *[5]. Adenovirus-mediated down-regulation of bicistronic constructs of uPA and uPAR expression inhibited cell migration, invasion and tumor-induced capillary formation[4]. In another study, stably transfected glioma cells expressing the amino terminal fragment (ATF) domain (residues 1–46) of uPA, which binds uPAR, did not form tumors in nude mice[6]. However, studies have demonstrated that both uPA^-/- ^and uPAR^-/- ^homozygous deficient mice develop normally without any apparent growth defect [7,8]. Therefore, targeting and inhibiting the uPA/uPAR system for cancer therapy is not likely to cause deleterious effects on normal cells and would be an appropriate approach for adjuvant therapy.

Epidermal growth factor receptor (EGFR) is a transmembrane glycoprotein with specific tyrosine kinase activity, serving to regulate proliferation and differentiation of epidermal cells[9,10]. In human solid tumors, over-activation and/or dysregulation of EGFR promotes processes involved in tumor progression, including invasion, angiogenesis, metastasis, and resistance to anticancer treatment with blocking apoptosis [11-13]. Activation of this receptor actually leads to recruitment and phosphorylation by protein kinases (PKs) of several intracellularsubstrates, which, in turn, engage mitogenic signaling and other tumor-promoting activities. Therefore, over 20 years ago, EGFR signaling inhibition was proposed as a target for cancer therapy[14].

Both EGFR and uPAR receptors interact with each other at many levels[15]. Part of cellular signaling from uPAR appears to occur through EGFR transactivation [16,17]. Furthermore, abrogation of EGFR signaling in tumor model systems blocks uPAR-associated invasiveness through an extracellular matrix [18] and growth of tumors in animal models [16,19]. Thus, EGFR appears to be a necessary element for uPAR-mediated tumor progression. On the other hand, some workers have shown that uPAR is also necessary for EGF to induce proliferation of mouse embryonic cells and some cancer cells [20].

The development of EGFR kinase inhibitors was greeted with tremendous enthusiasm in the therapy of squamous cell carcinoma of the head and neck (SCCHN) based on the nearly universal expression of this receptor in this cancer, the negative prognostic associations with expression, and robust preclinical data[21]. Furthermore, clinical trials to date have demonstrated modest activity of these drugs as single agents with reproducible major response rates of 5% to 15% in SCCHN depending on agent, dose, and schedule. However, the biology of responsiveness to these agents remains unclear[21,22]. Therefore, the mechanisms involved in resistance against these inhibitors as well as their side-effects are still important problems in using these anti-cancer agents.

Gefitinib ("Iressa" or ZD1839, from AstraZeneca. Co.) is an orally active, selective EGFR tyrosine kinase inhibitor blocking signal transduction pathways implicated in the proliferation and survival of cancer cells[23]. Our study focused on relationship between status of uPAR and EGFR pathway in head and neck cancer cells treated by tyrosine-kinase inhibitor gefitinib. Our results showed how down-regulation of uPAR was associated with changed EGFR trafficking upon gefitinib treatment. They also suggested that combined uPAR antisense treatment with inhibition of EGFR activation might improve the efficiency of the EGFR-targeting anti-cancer therapy and could be considered as a new therapeutic strategy.

## Results

### uPAR antisense down-regulation and its effect on EGFR

**Figure 1:**
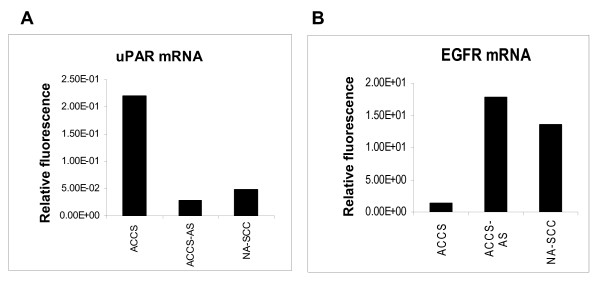
**Anti-sense induced down-regulation of uPAR was accompanied by an increased mRNA expression of EGFR n**: **(A) **RT- PCR confirmed the uPAR down-regulation in ACCS-AS cells and constitutively low uPAR expression in NA-SCC cells. **(B) **ACCS-AS cells showed marked increase in mRNA expression of EGFR, compared to the parental ACCS cells. Constitutive high expression of EGFR in NA-SCC cells is also shown.

uPAR antisense (AS) transfection of ACCS cells effectively down-regulated the mRNA expression of uPAR at least in five isolated ACCS-AS clones. uPAR expression in a representative clone is shown (Fig. 1,A). The uPAR levels in ACCS-AS cells reduced to the levels comparable to NA-SCC cell, a typical highly proliferative/less invasive squamous cell carcinoma (SCC) cell line of the head and neck, normally expressing low levels of uPAR (Fig. 1, A)(See also Additional file 1 for comparing the protein level).

ACCS generally showed low expression of EGFR (Fig. 1, B). However, antisense-induced uPAR down-regulation resulted in up-regulated expression of EGFR in ACCS-AS cells (Fig. 1, B). NA-SCC also showed constitutively high expression of EGFR mRNA (Fig. 1, B).

### Effect of Tyrosine kinase inhibition on cell proliferation

**Figure 2:**
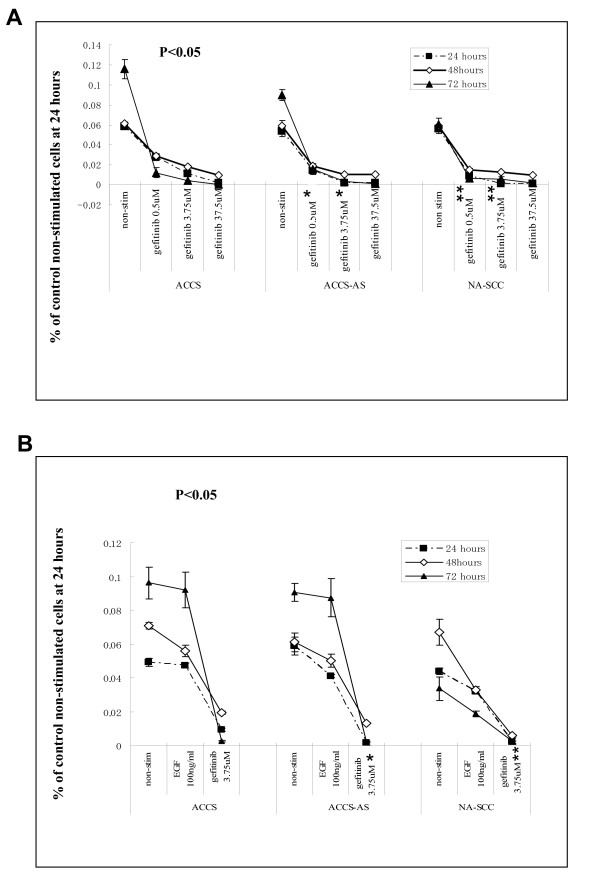
**The effect of Tyrosine kinase inhibition on cellular proliferation determined by MTT assay**: **(A) **the effects of increasing concentrations of gefitinib on growth of ACCS, ACCS-AS and NA-SCC cells are shown. Cells were incubated with vehicle, *i.e*., non-stimulated, or increasing concentration of gefitinib for 24, 48, and 72 hours. **(B) **Comparison of cellular growth in response to gefitinib (3.75 μM), EGF (100 ng/ml) and vehicle *i.e*., non-stimulated. The *bars *represent SD of results of separate wells. * p < 0.05, versus similar dosage in parental ACCS cells at 24 and 48 hrs. ** p < 0.05, versus similar dosage in ACCS cells at 24 and 48 hrs.

To demonstrate whether inhibition or stimulation of increased EGFR in uPAR down-regulated cells has any effect on their growth, we evaluated the cellular proliferation in uPAR down-regulated ACCS-AS cells and compared the results with parental ACCS and NA-SCC cells. We used MTT assay to evaluate the cellular proliferation and measured the combined effect of uPAR down-regulation and gefitinib treatment on growth potential of the cells in vitro. The proliferation rate was compared to EGF-stimulated or non-stimulated cellular proliferation (Fig. 2). MTT assay showed that although gefitinib inhibited cell proliferation and resulted in cell death in all examined cells, the intensity of response was not same among the cells. In fact, gefitinib inhibited cellular proliferation almost equally in high EGFR/low uPAR expressing NA-SCC and ACCS-AS cells at 0.5 *μ*M (Fig. 2, A). However, in parental ACCS cells, reversely expressing high uPAR/low EGFR profile, the effect was significantly lower (p < 0.05) and 7–10 times higher doses were required to achieve similar effects. Comparing the effects of EGFR stimulation by EGF and its inhibition by gefitinib showed that although EGFR inhibition had anti- proliferative effect, the EGF-induced stimulation of EGFR did not significantly alter total cellular proliferation in the examined cells (Fig 2, B).

### Tyrosine kinase inhibition reduced cell adhesion and chemotactic migration

**Figure 3:**
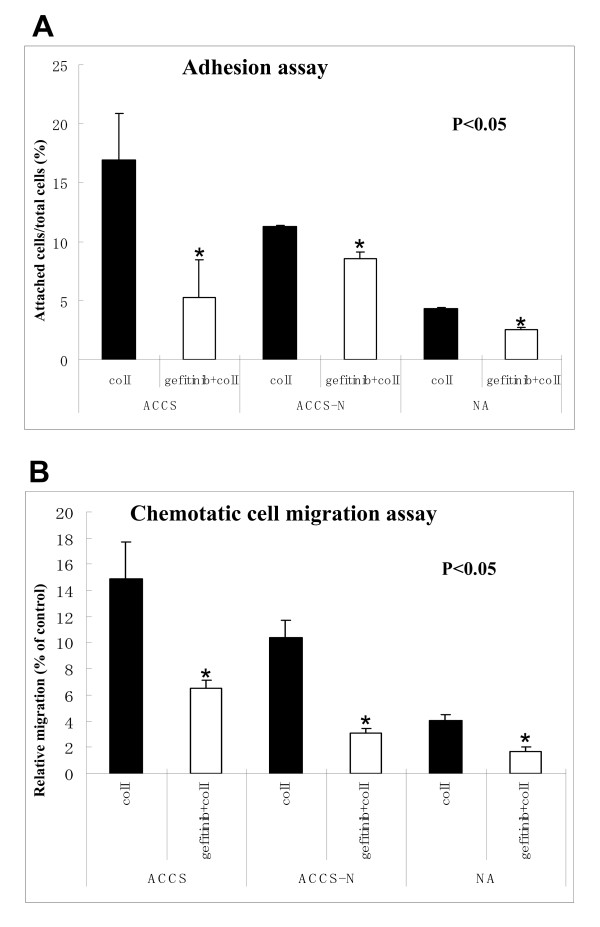
**The effect of Tyrosine kinase inhibition on cell adhesion and chemotactic migration**: The effect of gefitinib on cell adhesion **(A) **and chemotactic migration **(B) **was evaluated on collagen I as ECM. Gefitinib reduced both cell adhesion and chemotactic cell migration in the three cell lines. The inhibitory effect of gefitinib on chemotactic cell migration was more significant in ACCS-AS cells, compared to parental ACCS cells, suggesting a synergism between the dual inhibition of uPAR and EGFR signaling on cellular migration. The low adhesion and chemotactic migration of NA-SCC cells are also shown. The *bars *represent SD of results of separate wells. *p < 0.05, non-treated versus gefitinib-treated cells.

We have previously demonstrated that uPAR antisense treatment reduced the cell adhesion on various extracellular matrices (ECMs) [24]. In this study, we investigated the effect of gefitinib on the cell adhesion and chemotactic migration upon same ECM,*i.e*., collagen I (Fig. 3). Cellular adhesion in non-treated ACCS-AS cells was comparatively lower than the parental ACCS. NA-SCC cells showed the lowest adhesion. Gefitinib showed an inhibitory effect on the cellular adhesion in all cells (Fig. 3, A). However, comparing to non-treated cells, inhibitory effect of gefitinib on adhesion was not different between parental ACCS and ACCS-AS cells.

Cellular chemotactic migration was also evaluated using modified Boyden chamber as it is described in methods. Chemotactic migration in non-treated ACCS-AS cells was also comparatively lower than parental ACCS. NA-SCC cells showed the lowest migration. Gefitinib also affected cell chemotactic migration. In fact, gefitinib inhibited cell chemotactic migration over collagen I in all of examined cells. However, the reduced chemotactic migration was more significant in ACCS-AS cell line, compared with ACCS (p < 0.05)(Fig. 3, B).

### Tyrosine kinase inhibition affected the activation of EGFR and its downstream molecules

**Figure 4:**
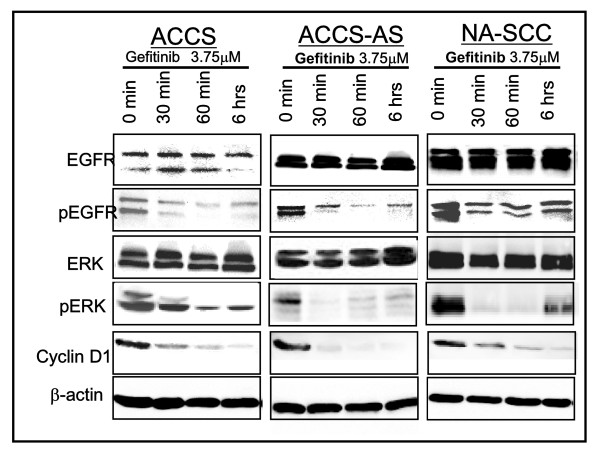
**The effect of Tyrosine-Kinase inhibition on activation of EGFR and its down-stream signaling molecules**: Gefitinib inhibited the phosphorylation of EGFR and ERK and reduced Cyclin D1 expression in all cells. Total EGFR and total ERK are also shown for comparison. β-actin western blots and Coomassie blue staining of gels were used for confirmation of equal loading.

Increased expression of EGFR in uPAR anti-sense transfected cells indicated a significant change in metabolism of EGFR. Therefore, next we studied the activation status of EGFR in the parental ACCS and uPAR down-regulated ACCS-AS cells showing increased EGFR expression. Activation status of EGFR in NA-SCC cells was also investigated for comparison (Fig 4). Gefitinib de-activated the auto-activated EGFR in the cells (Fig. 4). This de-activation was especially noted in ACCS-AS, as early as 30 minutes. However, in ACCS, the de-activation of EGFR was comparatively lower and later (60 minutes). In the NA-SCC cells also gefitinib effectively de-activated the EGFR as early as 30 minutes.

ERK is a main EGFR downstream signal mediator commonly evaluated after EGFR-targeted therapy. To investigate whether gefitinib affect the downstream mediators of EGFR in ACCS-AS cells with increased EGFR expression, the activation status of ERK pathway was also studied. The results showed that tyrosine kinase inhibition by gefitinib was associated with de-activation of ERK. Gefitinib actually affected ERK activation almost in similar pattern as EGFR (Fig. 4). Furthermore, comparing to ACCS, the de-activation in ACCS-AS cells was comparatively more significant.

Cyclin D1 expression is also linked to the stimulation or inhibition of growth factors-related signals including EGFR[25]. The Cyclin D1 expression was studied by immunoblotting. Cyclin D1 expression was also reduced in the three cell lines upon gefitinib treatment indicating effective inhibition of EGFR (Fig. 4).

### Morphological changes upon inhibition of EGFR activation

**Figure 5:**
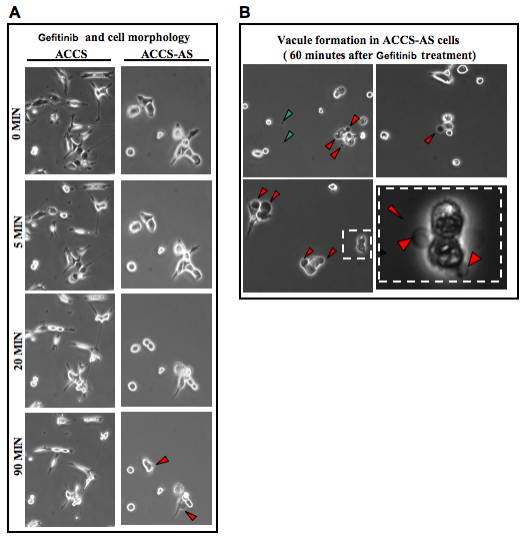
**Formation and exocytosis of vacuole-like structures upon inhibition of EGFR activation in uPAR down-regulated cells**: **(A) **Gefitinib led to reduced spreading and cell rounding which was more obvious in ACCS-AS cells. **(B) **There was also vacuolar formation observed as early as 10 minutes in ACCS-AS cell lines which continued and affected most of cell population with time. Representative vacuole formations on ACCS-AS cells (red arrowheads) are shown. Time-lapse videography also showed that formed vacuoles were exocytosed (see supplemented data). An example of masses speculated to be already exocytosed vacuole sacs are shown (green arrowheads).

Our previous study showed that uPAR down-regulation was associated with some morphologic changes in the cells plated on various ECMs, especially regarding their cytoplasmic projections [24]. In this study, the effects of Tyrosine kinase inhibition on cellular morphology were evaluated (Fig. 5). Gefitinib had a striking effect on cellular morphology. In fact, gefitinib treatment resulted in cell rounding and loss of pseudopodia which was more marked in ACCS-AS cells, compared with parental ACCS cells (Fig. 5, A). Using time-lapse videography, we actually observed the detailed events of cellular rounding of the cells upon gefitinib treatment [Additional file 2, 3 and 4]. The representative photomicrographs taken at 0, 5, 20, and 90 minutes are shown (Fig. 5, A). This was observed earlier in single cells. Then cells in cluster rounded consequently and exited from cell- cell junctions (Fig. 5, A, red arrowheads).

Cellular rounding was followed by formation of vacuole-like structures especially in ACCS-AS cells (Fig. 5, B). The appearance of dark vacuoles in ACCS-AS cells was documented with time-laps videography showing the detailed process of vacuole formation [Additional file 2, 3 and 4]. The formed vacuoles seemed to leave the cells as exocytosis sacs in these cells [Fig. 5, B and Additional file 2, 3]. NA-SCC cells with high EGFR/low uPAR profile also showed similar morphological findings to ACCS-AS cells [Additional file 4]. In contrast, the time-laps videography did not show any obvious evidence indicating formation of such vacuoles in ACCS cells.

### The vacuole-like structure contained de-activated EGFR

**Figure 6:**
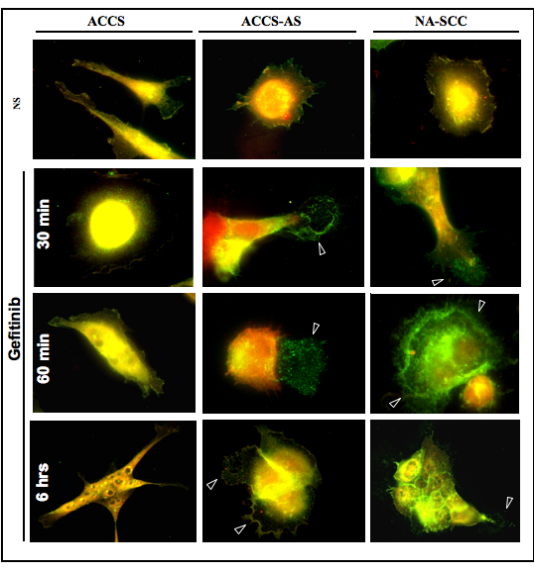
**De-activated EGFR constitutes the main component of the vacuole-like structure in ACCS-AS**: Cells were double-stained with anti-EGFR (green) and anti-phosphorylated EGFR(red). Immunocytochemistry showed that vacuole-like structure formed in ACCS-AS cells upon gefitinib treatment contained mostly de-activated EGFR. Attached cells were markedly reduced at 6 hours. In ACCS cells, vacuole-like formations were not detected. At 6 hours, the number of attached cells was also reduced. In ACCS cells, there were some evidences of internalization of EGFR to the cytoplasm.

Next we studied the nature and contents of vacuole-like structures. Immunocytochemistry with anti-EGFR showed that these vacuoles actually contained EGFR. Furthermore, double staining of the cells with anti-EGFR (FITC's green signal) and anti-phosphorylated EGFR,*i.e*., activated EGFR (TRITC's red signals) showed that in ACCS-AS and NA-SCC cells the observed vacuole-like structure contained only de-activated EGFR (the green mass lacking the red signal, arrowheads, Fig 6). Results also suggested that in ACCS cells, where EGFR expression is low, EGFR clustering at focal adhesions was low (Fig 6). In these cells, EGFR might either internalize into the cells (Fig. 6, ACCS at 6 hours) and/or small undetectable vacuole-like structures might be formed and exocytosed. However, in ACCS-AS, EGFR clusters were formed as early as 30 minutes, increased in size and formed marginal vacuoles then were expelled. At 6 hours, gradual loss of attached viable cells was observed with evidence of new EGFR clustering at the rims of still viable attached cells (Fig 6).

## Discussion

In this study, we have investigated the effects of a combination of tyrosine kinase inhibition of EGFR and uPAR down-regulation on EGFR signaling pathway in adenoid Cystic Carcinoma (ACC) cell lines. Then, the behaviors of uPAR down-regulated cells were compared with NA-SCC squamous carcinoma cells showing similar uPAR/EGFR expression profile.

Molecular inhibition of EGFR/HER1 signaling is extensively investigated as a promising cancer treatment strategy[26]. The potential value of modulating EGFR signaling as a cancer treatment approach is reflected by the broad array of molecular inhibitors that have been developed and launched in clinical trials during recent years[27]. The majority of patients on clinical trials with anti-EGFR agents have been enrolled within the past 3–5 years, and several dozen large-scale trials remain in progress or in final design. Despite broad enthusiasm regarding the potential value of EGFR target modulation in cancer therapy, the field rests at an important crossroads in light of negative results from several large-scale phase III clinical trials in lung cancer reported during 2002–2003 [28,29].

The side effects of EGFR tyrosine kinase inhibitors are important challenges in their usage in cancer therapy. In fact, inappropriate dosing could be the cause for the elevated risk of toxicity[30,31] and one report, released in 2003, has claimed that 246 patients actually have died from treatment itself [32].

For these reasons, recently, some authors have suggested the value of Dual-Agent Molecular Targeting of the EGFR[33]. They suggest that combined treatment with distinct EGFR inhibitory agents can augment the potency of EGFR signaling inhibition[33]. This approach suggests potential new strategies to maximize effective target inhibition, which may improve the therapeutic ratio for anti-EGFR-targeted therapies in developing clinical trials.

On the other hand, recent studies suggested that uPAR inhibition by RNA interference may be of great value for curbing the growth and spread of cancers [34]. As mentioned previously, based on data from knock-out mice studies, uPAR is not significantly important for the normal development and growth [7,8]. In fact, uPAR is rarely expressed on the surface of normal cells and mostly is considered as a specific marker for cellular malignant changes. For these reasons, it is targeted for cancer therapy by some researchers[35,36].

We have established antisense induced down-regulation of uPAR in ACCS cell lines (ACCS-AS)[24]. uPAR down-regulation resulted in decreased adhesion, migration and invasion on various ECMs [24]. In this paper we have shown the results on collagen I (Fig. 2). A recent study on osteosarcoma clones exhibiting uPAR down-regulation also demonstrated decreased adhesion, migration and invasion in cell-based assays in vitro[3]. In vivo study also showed that injection of mice with more potent antisense clones could completely prevent the pulmonary metastasis in the mice model [3]. The results of other studies working on glioblastoma also showed that cellular proliferation of cells was not affected by uPAR down-regulation[5,5].

uPAR antisense treatment led to an increase in EGFR expression in ACCS-AS cells(Fig. 1, B). This was in agreement with previous studies on HEp3 cell line[16]. The increased EGFR in uPAR-down-regulated cells led us to further investigate whether gefitinib as an EGFR inhibitory agent has any effect on the behavior of our established transfected cells. MTT assay showed the inhibitory effects of gefitinib on cellular growth (Fig. 2). Interestingly, although gefitinib inhibited the cellular proliferation, EGF did not increase it (Fig. 2). Lack of EGF-induced cellular proliferation has been reported since two decades ago [37]. In fact, EGF has been reported to both increase and decrease proliferation [38]. It has been shown that EGF inhibited cell proliferation and induced morphological features in some cells. It is believed that in cells with over-expressed EGFR, adding exogenous EGF induce apoptosis by induction of dramatic increase in EGF-R auto-phosphorylation, which down-regulate EGF signal transduction[39].

We showed that gefitinib de-phosphorylated EGFR and ERK (Fig. 4). This was comparatively more intensive and happened earlier in ACCS-AS cells, compared to parental ACCS (Fig. 4). Delayed and less intensive in-activation of ERK in ACCS could be attributed to uPAR-induced activation of ERK which is significantly suppressed in uPAR down-regulated ACCS-AS [40].

Furthermore, as results of this study showed, EGFR accumulated at focal adhesions upon de-activation by gefitinib (Fig 6). Localization of EGFR especially after bindings of ligands is an interesting part of EGFR signaling. In many cells, EGFR is found in caveolae [41]. Recently it has been established that upon EGFR activation, EGFR clusters dimerize and internalize [41-43]. Ligand-induced internalization of EGFR is suggested to follow the migration of active receptors out of caveolae, and subsequent receptor clustering over clathrin-coated vesicles [44,45]. These steps are suggested to enable the loading of monoubiquitylated receptors onto an ubiquitin-bound multimolecular complex that sorts ERBB1 in early endosomes. EGFR is further processed to luminal vesicles of the pre-lysosomal compartment [45,41]. In general, ligand-induced receptor endocytosis down-regulates growth factor signaling. However, internalized EGFR molecules are enzymatically active, hyperphosphorylated and associated with Shc, GRB2 and SOS[46] and endosomal EGFR signaling is sufficient to activate the main signaling pathways leading to cell proliferation and survival [42].

Interestingly, in this study, we observed a specific pattern of EGFR localization. In fact, videography showed a gradual appearance of vacuole-like structures upon gefitinib treatment in ACCS-AS (Fig. 5) as well as NA-SCC cells (Data not shown). Time-laps videos also showed that these vacuole-like structures were gradually undergoing exocytosis in ACCS-AS and NA-SCC cells [Additional file 2, 3 and 4]. In ACCS, however, there was no significant evidence of such vacuole formation. Interestingly, immunocytochemical study showed that these vacuole-like structures actually contained accumulated de-activated EGFR (Fig. 6, red arrow heads). Furthermore, our results suggested that upon gefitinib inhibition of EGFR activation in high EGFR/low uPAR cells, de-activated EGFR clustered (Fig 6). These clusters apparently failed to internalize. We speculate that the phenomenon might be either due to accumulation of de-activated EGFR at focal adhesion preventing their internalization or lowered uPAR expression somehow affected mechanisms controlling EGFR trafficking. However, in ACCS cell line, with high uPAR/low EGFR de-activated EGFR might cluster and internalize in endosomes as shown by immunocytochemistry at 6 hours (Fig. 6).

Finally, considering the effect on adhesion and chemotactic cell migration and proliferation, these results suggest that targeting both EGFR and uPAR might provide an alternative method on cancer control by inhibiting both its growth and metastasis. Furthermore, increased sensitivity of ACCS-AS cells to anti-tumor effects of lower doses of tyrosine kinase inhibitors suggested that by inhibiting both EGFR and uPAR, we might achieve further goals by reducing unwanted side effects resulted from high doses of such agents.

## Materials and methods

### Cells and culture

This study examined three human cell lines: ACCS [47-49] and its stabilized uPAR antisense transfected clones; *i.e*. ACCS-AS cells, as described previously [24] and NA-SCC cells [49] isolated from tongue squamous cell carcinoma. The cells were maintained in Dulbecco's modified Eagle's medium (DMEM) supplemented with 10% fetal bovine serum (FBS) and 2 mM L-glutamine in a 5% CO2 incubator at 37°C. The antisense transfected cells were maintained by Zeocin 100 ug/ml DMEM.

### Total RNA preparation and real-time RT-PCR

Total RNA was extracted from cultured cells using Sigma RNA-Easy kit, according to the manufacturer's protocol. First-strand cDNA was synthesized from 3 μg of total RNA using Superscript II reverse transcriptase (Life Technologies, Inc., Karlsruhe, Germany) and random hexanucleotide primers. The mRNA level of EGFR and uPAR was quantified using real-time PCR. Real-time monitoring of PCR products was performed by measuring the fluorescence (SYBR Green) of PCR products with the LightCycler (Roche Molecular Biochemicals, Mannheim, Germany). Normalization and quantification of RT-PCR were performed using Light Cycler software (Roche Molecular Biochemicals, Mannheim, Germany). The following sets of primers were used: uPAR, forward primer 5-TTACCTAATGCATTTCCT-3, reverse primer 5-TTGCACAGCCTCTTACCATA-3; β-actin(as internal control), forward primer 5-GTGGGGCGCCCCAGGCACCA-3, reverse primer 5-CTCCTTAATGTCACGCACGATTTC and EGFR, forward primer ^5'^GCGTCTCTTGCCGGAATGT^3'^, reverse primer ^5'^CTTGGCTCACCCTCCAGAAG^3'^

### Antibodies and reagents

Mouse anti-EGFR and anti-Phospho-EGFR were from BD Biosciences (BD Biosciences, San Diego, CA). Rabbit anti-EGFR was purchased from Chemicon (Chemicon, Temecula, CA). TRITC-conjugated mouse IgG1 antibody and FITC-conjugated anti rabbit IgG antibody were from Sigma (Sigma, St Louis, MO). Anti-ERK and anti-Phospho ERK antibodies were from Santa Cruz (Santa Cruz Biotechnology Inc., Santa Cruz, CA, USA). Anti-Cyclin D1 rabbit polyclonal antibody(#2922) was from Cell Signaling (Cell Signaling Technology, Beverly, MA, USA). Gefitinib (Iressa, ZD 1839) was obtained from AstraZeneca (AstraZeneCa Inc, UK).

### MTT Assay

The anti-proliferative activity of gefitinib was evaluated using an MTT-based assay as described previously[50]. Briefly, cells were harvested, washed with calcium- and magnesium-free PBS, re-suspended in DMEM and plated at a density of 1 × 10^4 ^vital cells into triplicate wells of flat bottom microtitration plates. Cells were allowed to adhere for 12 hours. Then fresh DMEM containing either DMSO or gefitinib at 0.5 uM; 3.75 uM; or 37.5 uM doses were added to determine the proper gefitinib dose. For the second step of MTT assay, serum-free DMEM containing 0.1%BSA, with either 100 ng/ml EGF, 3.75 μM gefitinib or DMSO as control were added. Incubation was done for 24, 48, and 72 h, and the number of metabolically active cells was determined. All experiments were repeated three times in triplets.

### Cell migration assay

A modified Boyden chamber was used for the cell chemotactic migration assay, as described previously [51] with brief modifications. The chamber (Falcon) consisted of upper and lower compartments separated by a polyethylene terephthalate track-etched filter (6.4 mm diameter) with 8 μm pores. The lower side of the filter was coated with collagen I or bovine serum albumin (BSA). Suspended cells (1 ~ 2 × 10^5 ^cells/well) in serum-free medium containing 0.3% BSA were placed in the upper compartment of the chamber and additional 100 ng/ml EGF, 3.75 μM gefitinib or DMSO (vehicle) were added. After 12 hours incubation at 37°C, migrated cells to the lower side of the filter were quantified and cells in five randomly chosen fields were counted. Counts were averaged and corrected with BSA cell invasion as control.

### Cell adhesion assay

Standard static adhesion assay was carried out as described previously [51] using a 96-well microtiter plate coated with collagen I or BSA. Briefly, 1~2 × 10^4 ^cells/well in serum-free DMEM containing 0.3% BSA, simultaneously with or without 3.75 uM gefitinib were added to the wells and allowed to adhere at 37°C for 60 min. Attached cells were fixed with methanol, stained with 0.5% crystal violet and lysed in 2% SDS. The absorbance was measured at 590 nm with a microplate reader.

### Evaluation of Morphology by Light Microscopy

Cells were harvested and plated in (Iwaki) non-coated dishes overnight. The next day, serum-free DMEM was aspirated and replaced with fresh serum-free DMEM with 3.75 μM gefitinib. Changes in cell morphology were examined under a Nikon Eclipse TE300 light microscope and photographed with a side attached Cool snap at determined times.

### Immunofluorescence staining

The cellular localization of focal adhesion components was analyzed using indirect Immunofluorescence staining. Cells were plated over cover-slips and incubated in serum-free DMEM for 12–16 hours then were treated with gefitinib at 3.75 μM. Cells were fixed in 4% Paraformaldehyde in BPS. Cover-slips were incubated with first antibodies for 1 h at room temperature, washed three times(each time for 10 minutes) with PBS, and then incubated with FITC-conjugated and/or TRITC-conjugated secondary antibodies. The images were captured using a CoolSNAP computer-controlled camera (Roper Scientific, Trenton, NJ) CCD camera and subsequently processed for analysis using Cool Snap software (Roper Scientific, Trenton, NJ).

### Immunoblotting analysis

Cells were harvested and plated in serum-free DMEM for 12–16 hrs then treated for 30, 60 min, and 6 hours with 3.75 μM gefitinib or non-stimulated(vehicle only). The cells were lysed with Ripa lysis buffer and insoluble material was pelleted at 14,000 rpm for 30 min at 4°C. Equal amounts of proteins were diluted in same Ripa buffer and sample buffer. The cell lysates were resolved by 10% SDS-PAGE, transferred to a nitrocellulose membrane, and incubated with specific primary antibodies. Protein bands were visualized using HRP-conjugated secondary antibodies and Enhanced Chemiluminescence Reagent (Amersham Pharmacia Biotech, Buckinghamshire, United Kingdom). Coomassie blue staining of the gels and immunoblotting with β-actin were used for confirmation of equal loading. The membranes were stripped using Restore™ Plus western blot stripping buffer (Thermo Scientific Inc., Rockford, IL, USA) blocked with skim milk and re-probed for other antibodies.

### Time-Lapse videography

Tissue culture plates were placed on the stage of a Nikon Eclipse 300 TE inverted microscope equipped with phase-contrast optics, a thermostatically controlled heating stage (Lincam Scientific Instruments, Surrey, UK), and a Plexiglas incubator (Nikon, Yokohama, Japan). The temperature inside the incubator was maintained at 37°C using a customized thermostatically controlled heating fan (DFA, Copenhagen, Denmark). To perform the videography, a CoolSNAP computer-controlled camera (Roper Scientific, Trenton, NJ) was mounted on the microscope.

### Statistical analysis

Comparisons between groups means were performed with paired, two-tailed Student's t test. P values less than 0.05 were considered statistically significant. Adhesion data were collected from triplicate experiments as the mean number of adherent cells in triplicate wells. The means ± SD represent the compiled data from experiments.

## Abbreviations

EGFR: epidermal growth factor receptor; uPAR: Urokinase Plasminogen Activator Receptor; ERK: extracellular signaling-related kinase; SCCHN: squamous cell carcinoma of the head and neck; ECM: extracellular matrices.

## Competing interests

The authors declare that they have no competing interests.

## Authors' contributions

SAA performed experiments and interpreted data; AF contributed to interpretation of data and preparation and organizing the manuscript. KS supervised all aspects of this research and preparation of the manuscript. All authors read and approved the final manuscript.

## Additional files

Cells were plated overnight in serum-free DMEM over non-coated dishes and left to adhere on their own ECM. On the next day, cells were washed with PBS and fresh serum-free DMEM was added to the culture plates. Culture plates were then placed under the microscope with incubator at 37°C, and 3.75 *μ*M gefitinib was added and videography was started.

## Supplementary Material

Additional file 1 uPAR antisense transfection of ACCS cells effectively down-regulated the protein expression of uPAR and up-regulated the EGFR in isolated ACCS-AS cells. Click here for file

Additional file 2 Time-lapse movie with 2 minutes intervals showed extensive rounding of ACCS-AS cells as early as 5 minutes upon gefitinib treatment. Cell rounding was followed by lost of intercellular contacts and vacuole-like structures formation. Click here for file

Additional file 3 Time-lapse movie with 5 minutes intervals, showed extensive rounding of ACCS-AS cells as early as 5 minutes upon gefitinib treatment and vacuole-like structures formations. Note: The Additional file 2 show that vacuole-like structure formed earlier in single cells. In Additional file 3, more single cells were observed in the field showing rounding and vacuole-like formation upon treatment with gefitinib. Cell detachment was also evident. Click here for file

Additional file 4 Time-lapse movie demonstrated that NA-SCC cells, expressing similar EGFR/uPAR receptor profile to ACCS-AS, also showed extensive rounding as early as 5 minutes upon gefitinib treatment as well as formation of vacuole-like structures that started soon after. Cell detachment was evident. Click here for file
