# Supplementary figures and images for: Tyrosine-kinase inhibition results in EGFR clustering at focal adhesions and consequent exocytosis in uPAR down-regulated cells of Head and Neck cancers
Source: Mol Cancer. 2008 Jun 3;7:47. doi: 10.1186/1476-4598-7-47 (PMC2464604; doi:10.1186/1476-4598-7-47)

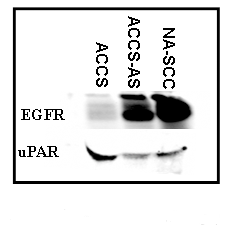

Supplement: Additional file 4 — Time-lapse movie demonstrated that NA-SCC cells, expressing similar EGFR/uPAR receptor profile to ACCS-AS, also showed extensive rounding as early as 5 minutes upon gefitinib treatment as well as formation of vacuole-like structures that started soon after. Cell detachment was evident. [file 1476-4598-7-47-S4.tiff]
